# Total body irradiation causes a chronic decrease in antioxidant levels

**DOI:** 10.1038/s41598-021-86187-1

**Published:** 2021-03-24

**Authors:** Lue Sun, Yohei Inaba, Yu Sogo, Atsuo Ito, Mahesh Bekal, Koichi Chida, Takashi Moritake

**Affiliations:** 1 Health and Medical Research Institute, Department of Life Science and Biotechnology, National Institute of Advanced Industrial Science and Technology (AIST), Central 6, 1-1-1 Higashi, Tsukuba, Ibaraki 305-8566 Japan; 2 Course of Radiological Technology, Health Sciences, Tohoku University Graduate School of Medicine, 2-1 Seiryo, Aoba, Sendai, Miyagi 980-8575 Japan; 3 Department of Radiation Disaster Medicine, International Research Institute of Disaster Science, Tohoku University, Aramaki Aza-Aoba 468-1, Aoba-ku, Sendai, 980-0845 Japan; 4 Department of Radiobiology and Hygiene Management, Institute of Industrial Ecological Sciences, University of Occupational and Environmental Health, Japan, 1-1 Iseigaoka, Yahatanishi-ku, Kitakyushu, Fukuoka 807-8555 Japan

**Keywords:** Mechanisms of disease, Senescence, Ecophysiology, Ageing, Biomarkers

## Abstract

Ionizing radiation exposure may not only cause acute radiation syndrome, but also an increased risk of late effects. It has been hypothesized that induction of chronic oxidative stress mediates the late effects of ionizing radiation. However, only a few reports have analyzed changes in long-term antioxidant capacity after irradiation in vivo. Our previous study demonstrated changes in whole-blood antioxidant capacity and red blood cell (RBC) glutathione levels within 50 days after total body irradiation (TBI). In this study, seven-week-old, male, C57BL/6J mice exposed to total body irradiation by X-ray and changes in whole-blood antioxidant capacity and RBC glutathione levels at ≥ 100 days after TBI were investigated. Whole-blood antioxidant capacity was chronically decreased in the 5-Gy group. The RBC reduced glutathione (GSH) level and the GSH/oxidative glutathione (GSSG) ratio were chronically decreased after ≥ 1 Gy of TBI. Interestingly, the complete blood counts (CBC) changed less with 1-Gy exposure, suggesting that GSH and the GSH/GSSG ratio were more sensitive radiation exposure markers than whole-blood antioxidant capacity and CBC counts. It has been reported that GSH depletion is one of the triggers leading to cataracts, hypertension, and atherosclerosis, and these diseases are also known as radiation-induced late effects. The present findings further suggest that chronic antioxidant reduction may contribute to the pathogenesis of late radiation effects.

## Introduction

Ionizing radiation exposure may not only cause acute radiation syndrome, but there is also an increased risk of late effects. It has been reported that the mortality or morbidity of cancer^1^, cataracts^2^, hypertension^3^, heart diseases^4^, atherosclerosis^5^, and strokes^4^ was increased in atomic bomb survivors a few decades after exposure. Most of these diseases are correlated with oxidative stress and persistent inflammation. Indeed, plasma reactive oxygen species (ROS), C-reactive protein (CRP), and interleukin-6 (IL-6) levels were increased in a dose-dependent manner in atomic bomb survivors^6^.

Antioxidants maintain redox homeostasis to maintain health by scavenging ROS and reducing oxidative metabolites and cytokines. We have analyzed changes in whole-blood antioxidant capacity from 30 min to 50 days after total body irradiation (TBI) in mice, finding that whole-blood antioxidant capacity decreased in a dose-dependent manner 2–24 days after TBI at 0.5–3 Gy^7^. Importantly, we also found that low antioxidant capacity persisted for at least 50 days after irradiation with TBI at 2 and 3 Gy^7^.

Glutathione is the most prevalent antioxidant in mammals, and the reduced glutathione (GSH)/oxidative glutathione (GSSG) ratio is considered a marker of redox homeostasis^8^. GSH also has anti-inflammatory activity. Depletion of GSH promotes or enhances oxidative stress^9^, inflammatory cytokine levels^10^, and radiation-induced cell damage^11^. Previously, we analyzed changes in red blood cell (RBC) glutathione levels from 2 to 24 days after TBI in mice, finding that radiation decreased the GSH/GSSG ratio through an increase of GSSG levels and a decrease of GSH levels^7^.

However, there is still a lack of information about whether whole-blood antioxidant capacity and RBC glutathione levels change 100 or more days after ionizing radiation exposure. This study extends our previous work and shows long-term changes in antioxidant levels following irradiation in vivo. Mice were exposed to TBI, and whole-blood antioxidant capacity, RBC glutathione levels, and complete blood counts (CBC) were examined every 100 days. It was found that whole-blood antioxidant capacity was chronically decreased in the 5-Gy group, and the RBC GSH level and the GSH/GSSG ratio were chronically decreased after ≥ 1 Gy of irradiation. These results suggest that radiation caused a lasting decline of antioxidant levels that may contribute to the pathogenesis of late effects.

## Results

### 3- and 5-Gy TBI shortened the life span

Seven-week-old, male, C57BL/6J mice received TBI with 0, 1, 3, and 5 Gy, and survival analysis was performed using the Kaplan–Meier method (Supplementary Fig. S1a). The median survivals in the 0-, 1-, 3-, and 5-Gy groups were 836.5 (95% confidence interval (CI) 880–804), 846 (95% CI 883–709), 659 (95% CI 520–743), and 609 (95% CI 531–734) days, respectively. The log-rank test was used to analyze the significance of differences in overall survival. The 1-Gy group did not show a shortened life span compared with the 0-Gy group (Supplementary Fig. S1b). The 3- and 5-Gy groups had significantly shorter survival than the 0-Gy group (Supplementary Fig. S1c,d).

### 5-Gy TBI chronically decreased whole-blood antioxidant capacity

**Figure 1:**
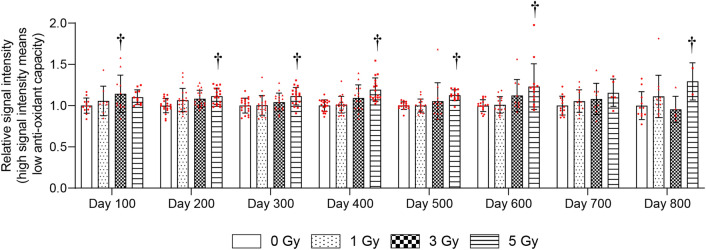
Change in antioxidant capacity of whole blood more than 100 days after total body irradiation (TBI). Whole blood antioxidant capacity was measured using i-STrap. Antioxidant capacity is inversely correlated with signal intensity. Bars indicate the means, error bars indicate the SDs, and red dots indicate individual data points. Two-way ANOVA and the post hoc Dunnett’s test were used to analyze significant differences with the 0-Gy group. ^†^Indicates P < 0.05.

The antioxidant capacity of whole blood was examined every 100 days after TBI with 1, 3, and 5 Gy. The 1-Gy group did not show significant changes in antioxidant capacity compared with the 0-Gy group (Fig. 1). The antioxidant capacity was significantly decreased at 100 days after 3-Gy, and 200–600 and 800 days after 5-Gy irradiation compared with the 0-Gy group (Fig. 1).

### TBI chronically decreased RBC glutathione levels

**Figure 2:**
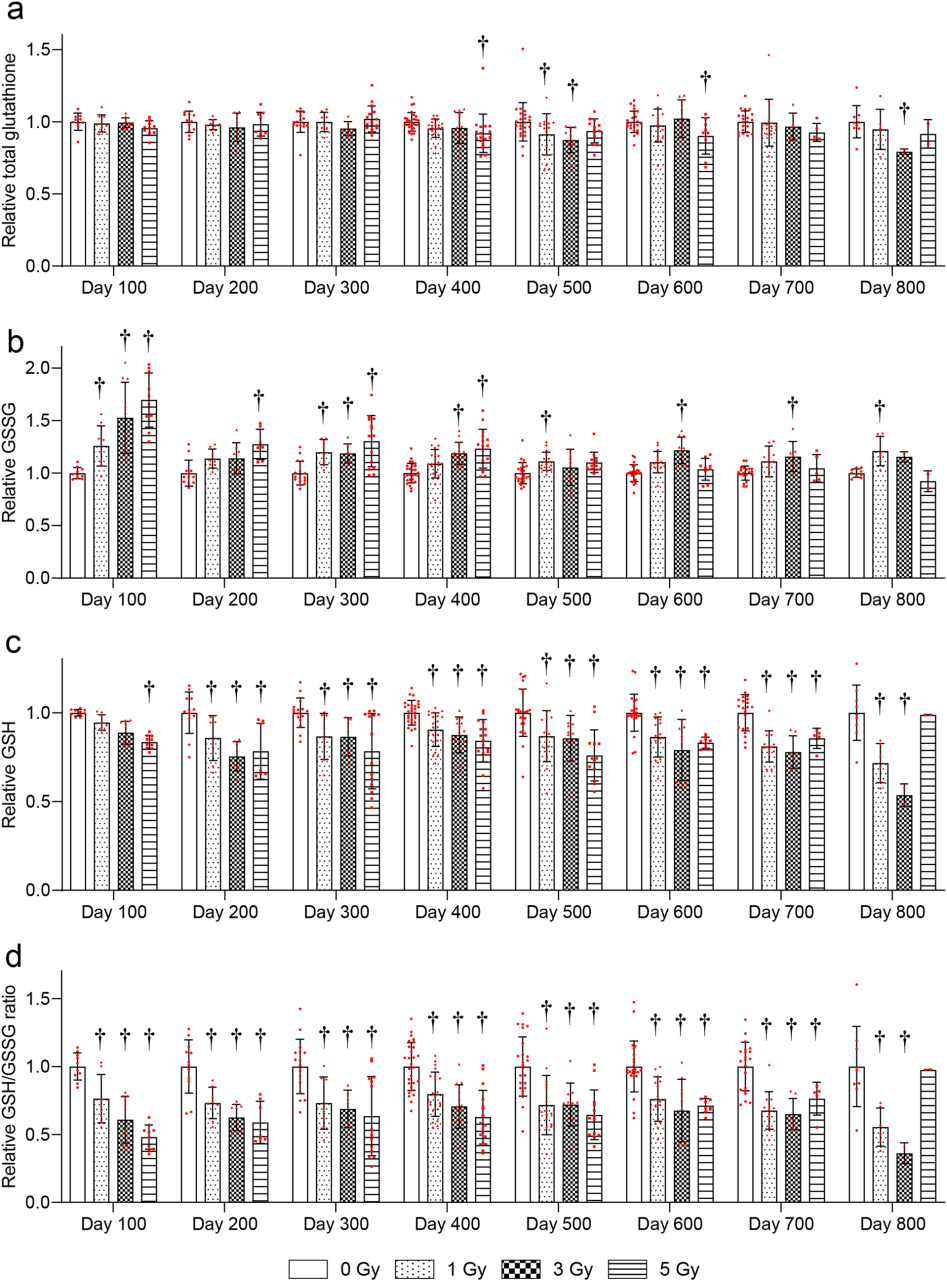
Changes in red blood cell (RBC) glutathione levels more than 100 days after TBI. Changes in (**a**) total glutathione, (**b**) reduced glutathione (GSH), (**c**) oxidized glutathione (GSSG), and (**d**) the GSH/GSSG ratio in RBC after irradiation. Bars indicate the means, error bars indicate the SDs, and red dots indicate individual data points. Two-way ANOVA and the post hoc Dunnett’s test were used to analyze significant differences with the 0-Gy group. ^†^Indicates P < 0.05.

The changes in RBC glutathione levels more than 100 days after irradiation were examined. The total glutathione levels were significantly decreased at 500 days after 1- and 3-Gy and at 400 and 600 days after 5-Gy irradiation compared with the 0-Gy group (Fig. 2a). GSSG levels were significantly increased at 100, 300, 500, and 800 days after 1-Gy, 100, 300, 400, 600, and 700 days after 3-Gy, and 100–400 days after 5-Gy irradiation compared with the 0-Gy group (Fig. 2b). GSH levels were significantly decreased at 200–800 days after 1- and 3-Gy, and 100–700 days after 5-Gy irradiation compared with the 0-Gy group (Fig. 2c). The GSH/GSSG ratio was significantly decreased at 100–800 days after 1-Gy, 100–800 days after 3-Gy, and 100–700 days after 5-Gy irradiation compared with the 0-Gy group (Fig. 2d).

### Changes in CBC after TBI

**Figure 3:**
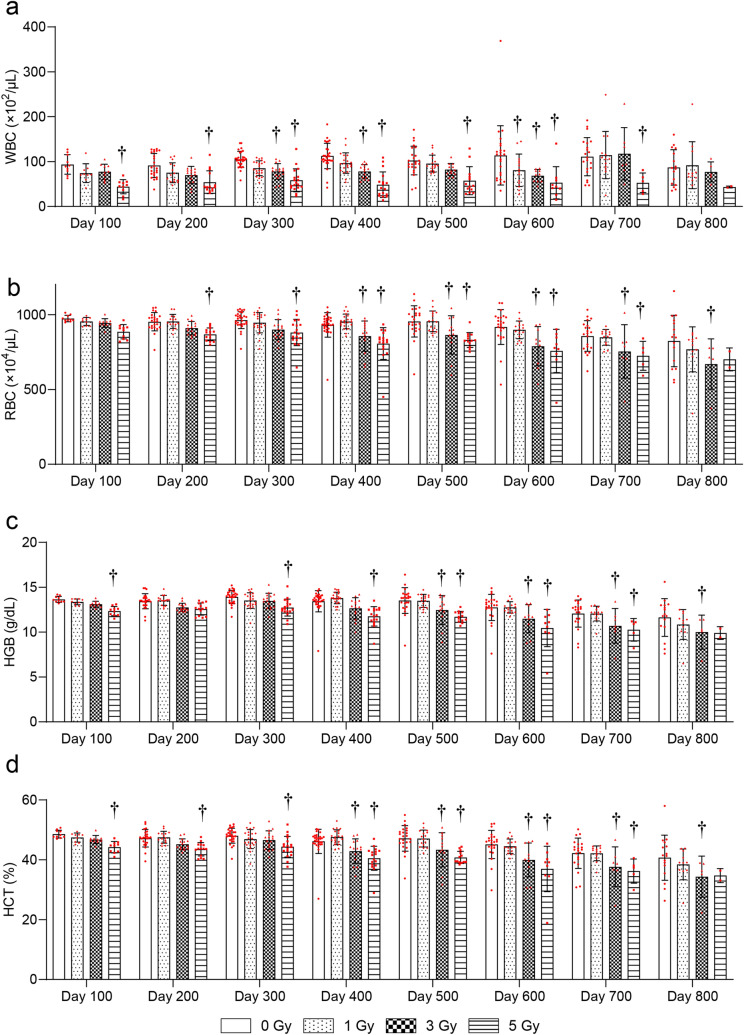
Changes in complete blood counts (CBC) more than 100 days after TBI. Changes in (**a**) white blood cell (WBC) counts, (**b**) red blood cell (RBC) counts, (**c**) hemoglobin (HGB), and (**d**) hematocrit (HCT). Bars indicate the means, error bars indicate the SDs, and red dots indicate individual data points. Two-way ANOVA and the post hoc Dunnett’s test were used to analyze significant differences with the 0-Gy group. ^†^Indicates P < 0.05.

**Figure 4:**
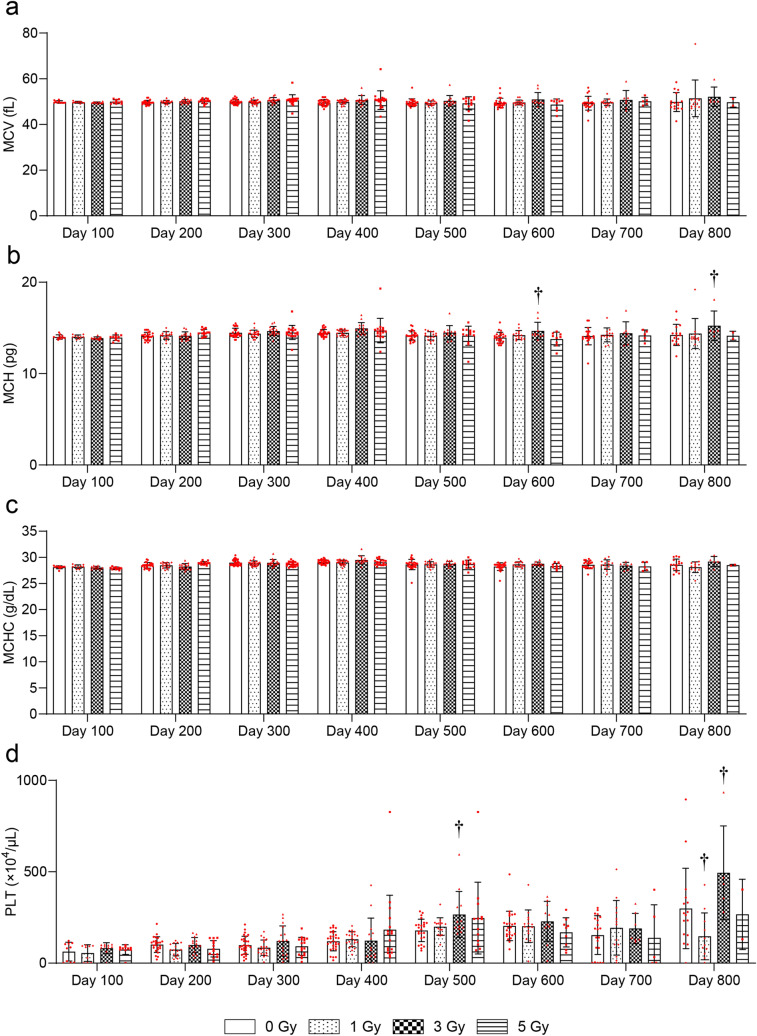
Changes in CBC more than 100 days after TBI. Changes in (**a**) mean corpuscular volume (MCV), (**b**) mean corpuscular hemoglobin (MCH), (**c**) mean corpuscular hemoglobin concentration (MCHC), and (**d**) platelets (PLT). Bars indicate the means, error bars indicate the SDs, and red dots indicate individual data points. Two-way ANOVA and the post hoc Dunnett’s test were used to analyze significant differences with the 0-Gy group. ^†^Indicates P < 0.05.

To evaluate radiation-induced blood injury, changes in the CBC later than 100 days after irradiation were evaluated. The white blood cell (WBC) count was significantly decreased at 600 days after 1-Gy, at 300, 400, and 600 days after 3-Gy, and at 100–700 days after 5-Gy irradiation compared with the 0-Gy group (Fig. 3a). The RBC count was significantly decreased at 400–700 days after 3-Gy and at 200–700 days after 5-Gy irradiation compared with the 0-Gy group (Fig. 3b). Hemoglobin (HGB) was significantly decreased at 500–800 days after 3-Gy and at 100 and 300–700 days after 5-Gy irradiation compared with the 0-Gy group (Fig. 3c). Hematocrit (HCT) was significantly decreased at 400–800 days after 3-Gy and 100–700 days after 5-Gy irradiation compared with the 0-Gy group (Fig. 3d). There were no significant radiation-related changes in mean corpuscular volume (MCV) at any time point (Fig. 4a). Mean corpuscular hemoglobin (MCH) was significantly increased at 600 and 800 days after 3-Gy irradiation compared with the 0-Gy group (Fig. 4b). There were no significant radiation-related changes in mean corpuscular hemoglobin concentration (MCHC) at any time point (Fig. 4c). Platelets (PLT) were significantly increased at 500 and 800 days after 3-Gy irradiation compared with the 0-Gy group (Fig. 4d).

## Discussion

The purpose of this study was to investigate long-term (over a year) changes in antioxidant levels following irradiation in vivo. Mice were exposed to acute TBI by X-ray at 1, 3, and 5 Gy. Acute exposure was assumed in atomic bombings or serious nuclear power plant accidents. Exposure to 1 Gy significantly increased the risk of non-cancer disease in atomic bomb survivors^12,13^. The half lethal dose within 30 days in humans was presumed to be ~ 4 Gy for acute TBI^14^. Furthermore, our preliminary experiment showed that most mice will be dead within 14 days after 7-Gy or higher irradiation. Thus, we analyzed this dose range in the present study.

The antioxidant levels were analyzed in blood, because hematopoiesis is one of the main vital processes in the body of mammals and is one of the most radiosensitive systems^15^. It was found that whole-blood antioxidant capacity decreased chronically in the 5-Gy group (Fig. 1). RBC GSH levels and the GSH/GSSG ratio were chronically decreased after ≥ 1-Gy irradiation (Fig. 2). Furthermore, ≥ 3-Gy irradiation decreased WBC and RBC counts, HGB, and HCT levels (Fig. 3). These results suggested that RBC glutathione levels may be the most sensitive long-term biomarker of radiation among these parameters.

It has been reported that buthionine sulfoximine (BSO), a GSH synthesis inhibitor, treatment induced or worsened radiation-related disease and health risk in mice, such as cataracts^16^, brain inflammation^10^, hypertension^17^, atherosclerosis^18^, decreased HDL levels^19^, increased oxidative DNA damage^20^, and tumorigenesis^21^. Thus, induction of radiation-related disease and health risk may occur through decreased GSH levels. It has been shown that BSO treatment decreased viability and proliferation of tumor cells in vitro and in vivo, suggesting that GSH is also essential in tumor growth^22,23^. Indeed, the present study showed that 1-Gy irradiation decreased GSH levels, but did not shorten the lifespan of C57BL/6J mice (Supplementary Fig. S1b). It has been reported that > 90% of irradiated C57BL/6J mice developed thymic lymphoma^24^. Richie et al. also reported that BSO treatment increased colon tumorigenesis, but it did not shorten mouse survival^21^. Thus, further studies should carefully analyze whether decreased GSH level increase cancer mortality in humans.

Radiation-induced long-term changes in oxidative stress levels and hematology findings have been studied in atomic bomb survivors. They received ~  < 3-Gy acute TBI^12^. Thus, the exposure situation is similar to that of the present study. However, no studies analyzed antioxidant levels in atomic bomb survivors. Hayashi et al. reported that plasma ROS levels were increased in a dose-dependent manner in atomic bomb survivors at ~ 50 years after exposure^6^. This result was concordant with the present results and suggested that chronic oxidative stress was increased after exposure. It has been reported that WBC counts were increased^25^, and HGB levels^26^ and RBC counts^27^ were decreased in atomic bomb survivors. WBC counts were inconsistent, but RBC counts and HGB levels were consistent with the present study. Chua et al. reported that WBC, RBC, and PLT counts were chronically decreased in surviving mice after ~ 7.8-Gy TBI^28^. These results are consistent with the present study. They also suggested that reduced CBC were induced by hematopoietic stem and progenitor cell dysfunction after TBI^28^.

It has been suggested that radiotherapy induced late tissue damage associated with oxidative stress^29^. Robbins et al. reported that 8-hydroxy-2′-deoxyguanosine levels were continuously increased in kidneys over the 24-week experimental period in 10 Gy or higher acute partial-body irradiated rats^30^. Kang et al. reported that 15-Gy irradiation on mice lung increased malondialic acid levels in their lungs 15–20 weeks after irradiation^31^. Yin et al. reported that 18-Gy irradiation to dog lung increased ROS levels in lung tissue, but serum malondialdehyde (MDA) levels and reductase (superoxide dismutase and glutathione peroxidase) activities were not associated with radiation^32^. These reports are consistent with the present study and suggest that radiation affected the long-term redox state. However, these reports performed local irradiation as for radiotherapy, and doses were higher than in the present study. Further studies should examine whether progression of diseases or tissue damage is associated with imbalance of the redox state in our experimental situation.

Several papers have analyzed the redox state in chronic exposure. Volkova et al. analyzed the Scots pine that is widespread in the area contaminated by the Chernobyl accident, finding increases in the GSH/GSSG ratio and in MDA levels in the exposed group^33^. Urushihara et al. analyzed cattle within the ex-evacuation zone of the Fukushima Daiichi nuclear plant accident, finding increased glutathione peroxidase activity and MDA levels in the exposed group^34^. Malekirad et al. analyzed radiology staff, finding increased total antioxidant capacity and lipid peroxidation levels in the exposed group^35^. Thus, these reports are inconsistent with the present study and showed that chronic radiation exposure enhances both oxidative stress and antioxidants. These results suggest that dose rate and total dose are important factors in radiation-induced antioxidant level modification.

The present results leave several open questions. First, antioxidant levels were analyzed after acute TBI. However, induction of biological radiation effects varies with the exposure conditions (e.g. type of radiation, dose rate, irradiation volume, linear energy transfer, and total dose). Furthermore, TBI models do not completely mimic the nuclear disaster scenario or other uncontrolled nuclear events^28^. Future studies should analyze changes in the blood redox state in partial-body or chronic irradiation and determine whether the blood redox state is related to the progression of diseases. Second, young adult (7-week-old) male mice were used in the present study. It has been reported that biological radiation effects vary with age and sex^36,37^. Further studies should examine whether age and sex affect radiation-induced antioxidant changes. Third, the number of individual samples was decreased at ≥ 700 days, especially in the 3- and 5-Gy groups, which reduced statistical power. This was because most of the mice that received 3- and 5-Gy irradiation were dead before 700 days, and only limited breeding space was available. More importantly, it is necessary to consider survivorship bias in the ≥ 700-day groups.

## Conclusions

In conclusion, the present study showed that radiation has lasting effects on the antioxidant homeostasis of blood. Whole-blood antioxidant capacity and the RBC GSH/GSSG ratio were chronically decreased after TBI. Considering the present findings and those of previous studies, chronic antioxidant reduction may contribute to the pathogenesis of late radiation effects. Furthermore, the present results also support the hypothesis that the redox state could be a marker for estimating the risk of late radiation effects.

## Materials and methods

### Mice, irradiation, and blood sampling

Six-week-old, male, C57BL/6J mice were obtained from Japan SLC (Shizuoka, Japan). Their diet and drinking water were sterilized by autoclaving^7^. After at least 1 week of acclimation, the mice received TBI (0, 1, 3, and 5 Gy) using an X-ray generator (150 kV; 20 mA; filter: 0.2 mm Cu and 0.5 mm Al; MBR-1520R-3; Hitachi Power Solutions, Ibaraki, Japan. The dose rate was 0.69 Gy/min). Whole blood was collected by a 0.5-mm Goldenrod Animal Lancet (MEDIpoint, New York, NY, USA) puncture of the submandibular vein at 100, 200, 300, 400, 500, 600, 700, and 800 days after irradiation.

Whole blood was collected into heparin-containing tubes and centrifuged at 3000×g and 4 °C for 10 min to separate plasma and red blood cells^7^.

### Measurement of whole-blood antioxidant capacity (i-STrap)

Whole-blood antioxidant capacity was measured using the i-STrap technique (Dojindo/Dojin Glocal, Kumamoto, Japan), according to the manufacturer’s protocol^7^. Briefly, 100 μL of whole blood, 100 μL of saline, 10 mM of 2-diphenylphosphinoyl-2-methyl-3,4-dihy- dro-2H-pyrrole N-oxide (DPhPMPO), and 10 mM tert-butyl hydroperoxide (tBuOOH) were mixed and incubated at room temperature for 30 min^7^. Then, 1 mL of chloroform/methanol (2:1) solution (FUJIFILM Wako Pure Chemical Industries, Osaka, Japan) was added and mixed for 10 min. The samples were centrifuged at 8000×*g* and 4 °C for 10 min, and the organic layer was collected into a new tube and stored at − 80 °C until electron spin resonance (ESR) measurement^7^. The samples were measured by X-band ESR spectroscopy (JES-TE200; JEOL, Tokyo, Japan). The ESR conditions were as follows: microwave frequency, 9.423 GHz; microwave power, 2 mW; field center, 332.0 mT; sweep width, 20 mT; sweep time, 4 min; and time constant, 0.3 s. The signal of DPhPMPO spin adduct intensity was corrected by marker manganese oxide intensity^7^. The number of mice in each group is shown in Supplementary Table S1.

### Measurement of red blood cell glutathione levels

RBC glutathione levels were measured using a GSSG/GSH Quantification Kit (Dojindo) according to the manufacturer’s protocol^38^. Briefly, RBCs were hemolyzed with 10 times the amount of 5% 5-sulfosalicylic acid solution (FUJIFILM Wako), and the samples were centrifuged at 8000 × g for 10 min to remove proteins^7^. The samples and buffer solution were mixed and incubated for 1 h at 37 °C. Then Substrate and Enzyme/coenzyme working solution was added. After 10 min of incubation, absorbance was measured at 412 nm using a Varioskan LUX plate reader (Thermo Fisher Scientific, Kanagawa, Japan). The number of mice in each group is shown in Supplementary Table S1.

### Complete blood counts

Whole blood was collected into heparin-containing tubes. The samples were analyzed using a pocH-100iV instrument (Sysmex, Hyogo, Japan)^7^. The number of mice in each group is shown in Supplementary Table S1.

### Statistical analysis

The median survival time and 95% CI were calculated, and the significance of differences in overall survival was determined using the log-rank test. The mean and standard deviation (SD) values were calculated for the data of whole-blood antioxidant capacity, RBC glutathione levels, and CBC. Two-way ANOVA and the post hoc Dunnett’s test were used to analyze significant differences with the 0-Gy group. A P-value of less than 0.05 was considered significant.

### Ethical considerations

All animal experiments were performed in accordance with the ARRIVE guidelines, the Animal Care Guidelines of the University of Occupational and Environmental Health, Japan (UOEH.J.) and the National Institute of Advanced Industrial Science and Technology (AIST). All animal husbandry procedures and experiments were approved by the Animal Experiment Committee of UOEH.J (Permit Number: AE15-009) and AIST (Permit Number: 2019-0349).

## Supplementary information

Supplementary information.

## Data Availability

The authors declare that all data supporting the findings of this study are available within the paper.
