# Supplementary material for: Total body irradiation causes a chronic decrease in antioxidant levels
Source: Sci Rep. 2021 Mar 24;11:6716. doi: 10.1038/s41598-021-86187-1 (PMC7990969; doi:10.1038/s41598-021-86187-1)
Supplement: Supplementary file 1 — Supplementary information. [file 41598_2021_86187_MOESM1_ESM.pdf]

Total body irradiation causes a chronic decrease in antioxidant levels

Lue Sun[1)*], Yohei Inaba[2)3)], Yu Sogo[1)], Atsuo Ito[1)], Mahesh Bekal[4)], Koichi Chida[2)3)] and Takashi Moritake[4)**]

Affiliation:
1) Health and Medical Research Institute, Department of Life Science and Biotechnology, National Institute of Advanced Industrial Science and Technology (AIST), Central 6, 1-1-1 Higashi, Tsukuba, Ibaraki 305-8566, Japan.
2) Course of Radiological Technology, Health Sciences, Tohoku University Graduate School of Medicine, 2-1 Seiryo, Aoba, Sendai, Miyagi 980-8575, Japan.
3) Department of Radiation Disaster Medicine, International Research Institute of Disaster Science, Tohoku University, Aramaki Aza-Aoba 468-1, Aoba-ku, Sendai, 980-0845, Japan.
4) Department of Radiobiology and Hygiene Management, Institute of Industrial Ecological Sciences, University of Occupational and Environmental Health, Japan, 1-1 Iseigaoka, Yahatanishi-ku, Kitakyushu, Fukuoka 807-8555, Japan
* lue.sun@aist.go.jp
** moritake@med.uoeh-u.ac.jp

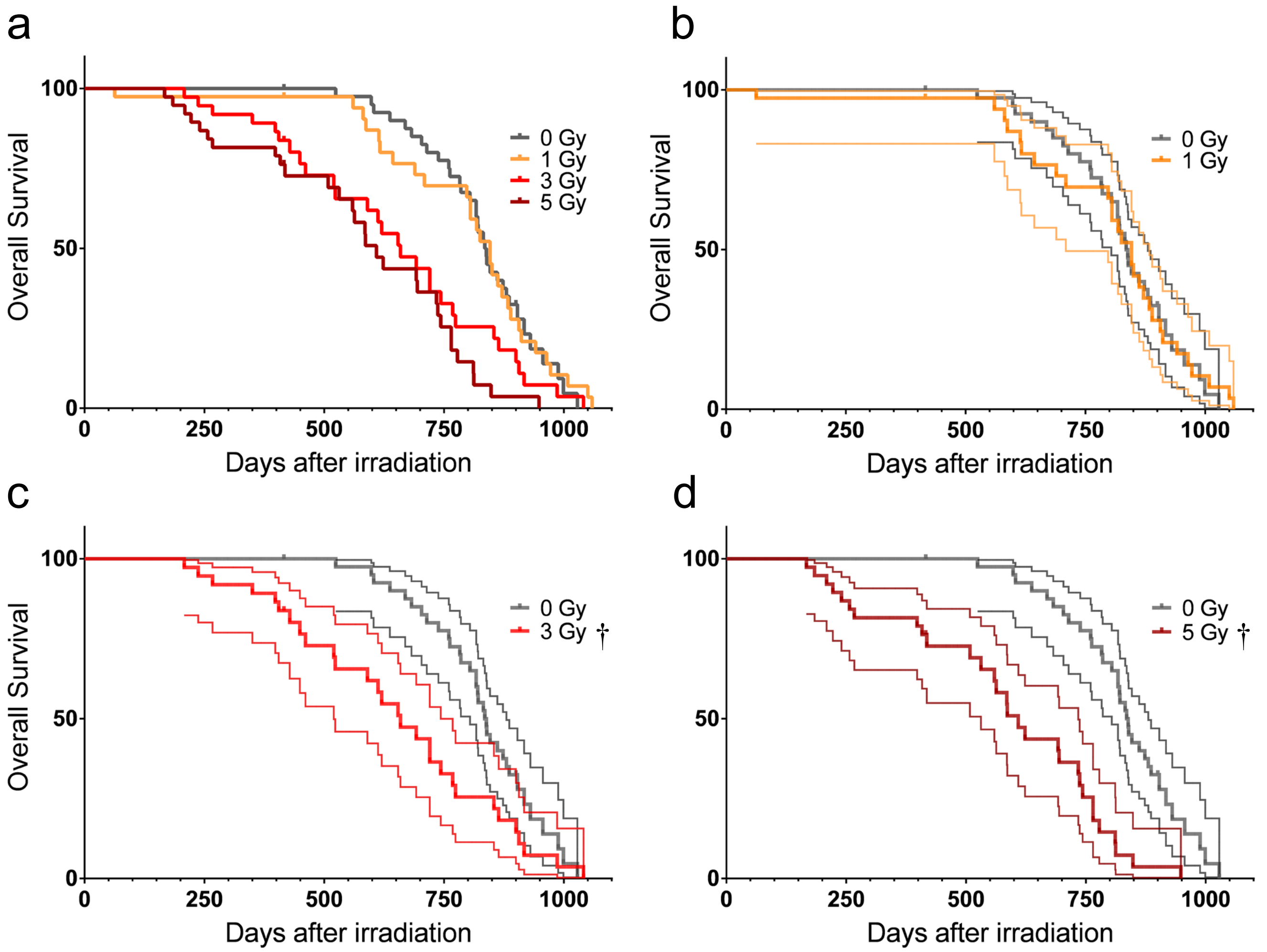

e

Number at risk.

Days after irradiation	0	250	500	750	1000
0 Gy	50	50	40	31	1
1 Gy	39	38	28	20	3
3 Gy	37	35	20	9	1
5 Gy	38	33	20	7	0

Supplementary Figure S1. Survival of irradiated mice. (a-d) Survival curves of the total body-irradiated mice (Thick lines) with 95% confidence intervals (Thin lines). (a) 0 Gy, 1 Gy, 3 Gy vs 5Gy, (b) 0 Gy vs 1 Gy, (c) 0 Gy vs 3 Gy, (d) 0 Gy vs 5 Gy. † indicates $P<0.05$, log-rank test, t-test.

Supplementary Table S1. Number of mice in each experiment.

	Day 100				Day 200				Day 300				Day 400			
	0 Gy	1 Gy	3 Gy	5 Gy	0 Gy	1 Gy	3 Gy	5 Gy	0 Gy	1 Gy	3 Gy	5 Gy	0 Gy	1 Gy	3 Gy	5 Gy
Whole-blood antioxidant capacity (i-STrap)	12	8	11	11	20	20	18	18	20	20	16	18	20	20	14	17
RBC glutathione levels	12	8	8	8	10	9	10	10	12	8	8	7	16	8	7	14
Complete blood counts	10	9	10	10	24	15	15	14	29	21	16	18	29	21	14	17

	Day 500				Day 600				Day 700				Day 800			
	0 Gy	1 Gy	3 Gy	5 Gy	0 Gy	1 Gy	3 Gy	5 Gy	0 Gy	1 Gy	3 Gy	5 Gy	0 Gy	1 Gy	3 Gy	5 Gy
Whole-blood antioxidant capacity (i-STrap)	16	16	12	13	16	15	10	13	14	12	8	5	11	11	5	2
RBC glutathione levels	28	21	14	16	24	16	11	13	24	15	10	9	21	13	7	5
Complete blood counts	24	16	12	13	24	14	10	9	21	13	7	5	16	11	5	2
